# Competency-Based Medical Education at Scale: A Road Map for Transforming National Systems of Postgraduate Medical Education

**DOI:** 10.5334/pme.957

**Published:** 2024-02-06

**Authors:** Jolanta Karpinski, Jennifer Stewart, Anna Oswald, Timothy R. Dalseg, Adelle Atkinson, Jason R. Frank

**Affiliations:** 1 Department of Medicine, University of Ottawa, Ottawa, ON, Canada; 2 Competency Based Medical Education, University of Ottawa, Ottawa, ON, Canada; 3 Royal College of Physicians and Surgeons of Canada, Ottawa, ON, Canada; 4 Specialty Standards, Office of Standards and Assessment, Royal College of Physicians and Surgeons of Canada, Ottawa, ON, Canada; 5 Division of Rheumatology, Department of Medicine, Faculty of Medicine and Dentistry, University of Alberta, Edmonton, AB, Canada; 6 Competency Based Medical Education, University of Alberta, Edmonton, AB, Canada; 7 Department of Medicine, Division of Emergency Medicine, University of Toronto, Toronto, ON, Canada; 8 Department of Paediatrics, Temerty Faculty of Medicine, University of Toronto, Toronto, ON, Canada; 9 Department of Emergency Medicine, and Director, Centre for Innovation in Medical Education, Faculty of Medicine, University of Ottawa, Ottawa, ON, Canada

## Abstract

In the past decade, the Canadian system of postgraduate medical education has been transformed with the implementation of a new approach to competency based medical education called Competence by Design. The Royal College of Physicians and Surgeons of Canada (Royal College) developed an approach to time-variable competency based medical education and adapted that design for medical, surgical, and diagnostic disciplines. New educational standards and entrustable professional activities consistent with this approach were co-created with 67 specialties and subspecialties, and implementation was scaled up across 17 universities and over 1000 postgraduate training programs. Partner engagement, systematic design of workshops to create discipline specific competency-based standards of education, and agile adaptation were all key ingredients for success. This paper describes the strategies applied by the Royal College, lessons learned regarding transformative change in the complex system of postgraduate medical education, and the current status of the Competence by Design initiative. The approach taken and lessons learned by the Royal College may be useful for other educators who are planning a transformation to CBME or any other major educational reform.

## Introduction

Since 1990, the Royal College of Physicians and Surgeons of Canada (hereafter referred to as the Royal College) has embarked on a deliberate path of evolving the Canadian system of specialty education toward competency based medical education (CBME). The Royal College oversees specialty education in 67 specialty and subspecialty disciplines (28 specialties, 37 subspecialties, and two special programs). Each discipline has a specialty committee to oversee the discipline at the national level; membership includes educational leaders, regional representatives, and all the postgraduate training program directors in the discipline. Working with the specialty committee, the Royal College sets educational standards for each discipline based on a common educational framework (CanMEDS) [1,2], sets standards for accreditation of individual postgraduate training programs, develops and delivers certification examinations, and credentials individual physicians. Postgraduate training programs are based in academic health science centres affiliated with a university faculty of medicine, overseen by a postgraduate medical education (PGME) dean and accredited by the Royal College. Canada has 17 faculties of medicine, approximately 1000 postgraduate training programs, and over 10,000 trainees.

Competence by Design (CBD) is a transformational change initiative in the system of Canadian specialty medical education designed to address societal health needs and patient outcomes [3]. It builds on previous Royal College approaches to CBME (e.g., the CanMEDS competencies) and applies the five core components of CBME [4]. Features of the CBD framework include its basis in the CanMEDS 2015 competency framework, a time-variable approach to postgraduate training, sequencing of training along the four stages of the Competence Continuum, and the use of entrustable professional activities (EPAs) specific to each stage (i.e., the Royal College approach to EPAs abbreviated as RCEPAs) as a focus for teaching, learning, observation, coaching, and assessment [5]. With the launch of CanMEDS 2015 in September 2015, the Royal College embarked on an ambitious initiative to apply the CBD framework to each of its specialties, subspecialties, and special programs.

## CBD Specialty Transformation

At the outset, the Royal College established several principles to guide the design and implementation of this change initiative. **There would be a common approach**; the educational design of CBD and its use of the five core components of CBME would apply to every specialty, subspecialty, and special program recognized by the Royal College. **Standards would be specific to each discipline**; within the framework of this educational design, each discipline would develop and individualize educational standards to fit the needs of the discipline. **The new CBD standards would be co-created by the discipline and the Royal College**, with the specialty committees providing medical and educational expertise and the Royal College providing resources, logistical support, and expertise in the CBD framework and in group facilitation. **The transformation would be iterative**; timely adaptations and modifications would be made to the design and process based on lessons learned.

To carry out this initiative, participants would need to develop new knowledge and skills about CBME and the CBD educational design, adapt and/or create new training standards and requirements within this paradigm, and lead or manage the change within their own spheres of responsibility. The CBD project team applied faculty development, curriculum design, and change management frameworks/models to develop a multi-year plan for CBD specialty transformation (see Table 1).

**Table 1:** Theoretical frameworks/models used in the approach to transform residency education towards a competency based medical education (CBME) model.


PURPOSE	FRAMEWORK/MODEL	ILLUSTRATION OF THE USE OF THE FRAMEWORK/MODEL

Preparation for Implementation	Change management/leadership using Kotter’s 8 steps and ADKAR [6,7]	Interactions with partners, including specialty committees, began with consulting about the need to change, establishing their support, and incorporating feedback into the vision for Competence by Design. Workshop participants developed their knowledge and skills about the change, applied that knowledge and skills to develop the educational design for that discipline, and participated in development activities at workshops and in webinars to support implementation of the educational design.

Participant development	Faculty development using Yvonne Steinert’s Core Concepts and Principles [8]	Workshops provided both formal and informal group learning: clinician educators provided information; the group work with clinician educator assistance provided informal group learning; development of communities of practice within the discipline through the shared activities provided informal group learning. Online modules provided formal individual online learning.

Standards development	Curriculum design using Expertise theory [9]	Standards development began with a discussion of the overall scope of practice, followed by identification of the tasks of the discipline which were used to identify the requirements for assessment of the development of competences as well as the required learning experiences.


## Engaging with Partners

Beginning in 2012, the CBD project team consulted with key partners in the PGME system about the proposed CBD framework and the plan for CBD implementation. The consultations included university PGME deans, the national specialty committees, national specialty societies, trainee organizations, relevant regulatory authorities, and Royal College clinician educators. The CBD project team leveraged longstanding relationships with these partners and regular (at least annual) interactions at meetings hosted by the Royal College or the respective organization. These discussions provided the project team with opportunities to inform and engage the PGME community while also gathering their input on the proposed initiative. In addition, this consultation identified specialty committees who were early adopters, that is those eager to start the transformation or already making progress toward the goals of CBD.

## Making the Road Map

The subsequent plan for CBD and its implementation addressed issues identified in the consultations with the above-named partners. Concerns had been raised about the workload involved, the availability of resources and support for the change, and the timelines associated with the initiative. Another common concern related to ongoing Royal College accreditation activities, including the workload for programs and schools associated with having to manage accreditation activities at the same time as CBD transformation while also meeting requirements for new accreditation standards related to the changing educational design. A challenging aspect of accreditation in relation to this curricular change was the mechanism of operation of Royal College accreditation activities. While accreditation is focused on one university, its PGME office, and all its postgraduate training programs, changes to educational standards occur at the level a discipline’s training programs across all universities. Thus, a university undergoing accreditation would have programs at varying stages in the CBD transformation process.

The road map for transformation was based on the principle that the Royal College would directly support the work of developing the specialty’s educational design (SED) and implementing CBD. This led to five key decisions:

1. The creation of new educational standards would be done by the Royal College specialty committee and would be used as the basis for simultaneous CBD implementation across all programs in that discipline.
2. Disciplines would be grouped into cohorts; each cohort would begin the work in sequence rather than all at once.
3. The grouping and sequencing of disciplines within cohorts would be intentional, based on the discipline’s preference, category (surgical, medical, laboratory, specialty, subspecialty, etc.) and relationship to other disciplines (see Supplementary file 1: Sequence and grouping of the cohorts).
4. The Royal College would establish and support a team of clinician educators, writers, and administrators that would work with each individual specialty committee to create their SED.
5. The work would occur through a series of workshops funded and supported by the Royal College.

The aforementioned concerns about workload and resources drove the decisions regarding centralized support for the specialty committee and funding for the series of workshops. The Royal College committed to facilitate the work of each specialty committee as they adapted the overarching framework of CBD for their own discipline. Through this work, program directors would have direct input into the specialty’s educational design, would engage in faculty development, and would be provided with the curricular elements that guide the sequence of training (i.e., stages, RCEPAs, assessment strategies, and training experiences) in addition to the final graduating set of discipline-specific competencies. By centralizing this work and providing financial support as well as logistical and CBD expertise, the Royal College aimed to reduce the burden on individual schools, programs, and program directors.

The decision to proceed with a cohort approach was pragmatic. With a commitment to support the specialty committees with funding and expertise, it was not feasible to work with all 67 committees simultaneously. The sequential engagement with different disciplines spread the financial impact over a decade. It also limited the number of disciplines actively engaged in the workshops or in CBD implementation at any one time, which enabled the Royal College to target support and other resources (the clinician educators, the education writers, faculty development support, etc.) to those disciplines. The specialty committees were polled to determine their preference for when to initiate the work, and factors such as their preference and the specialty committee’s workload related to other Royal College commitments were considered in their placement within a cohort.

The grouping and sequencing of disciplines within cohorts was also strategic. The Royal College intentionally placed “early adopters” in the first two cohorts; these disciplines had already participated in some preparation for or transformation to a CBME approach (e.g., by creating EPAs or developing a curriculum for simulation-based training and assessment). The first two cohorts also intentionally included a representative mix of Royal College disciplines: they included specialties and subspecialties; medical, surgical, and diagnostic disciplines; and disciplines with varying durations of postgraduate training (one to five years) (see Supplementary file 1). One of the goals of this careful assignment was to examine whether the uniform design of CBD could be applied to a variety of disciplines. The relationship of disciplines to each other was another factor in the strategic sequencing; for example, Obstetrics and Gynecology was sequenced to begin the CBD transformation before its three subspecialties, which were sequenced to begin at the same time as each other. It was perceived that this sequencing by “families” of disciplines could facilitate the work of CBD implementation by engaging a larger group of faculty members in that field before the smaller group of subspecialists.

The series of workshops was designed to sequence the work of creating new educational standards and break it down into manageable “chunks” of time and effort, thus facilitating specialty committee participation and minimizing committee members’ time away from clinical and other responsibilities. The workshops were a series of 3 three-day sessions held at approximately six-month intervals. Each workshop balanced work on the standards, faculty development, and preparation for CBD implementation. The entire specialty committee was funded to attend the full series of workshops, and each committee was encouraged to include one or two trainees as well as one to three other guest contributors (e.g., a member of the discipline with expertise in CBME or work-based assessment). Royal College clinician educators facilitated the workshops: they explained CBME, led the specialty committee through the sequence of workshop activities, and guided them through the CBD framework. The Royal College Specialties Unit organized the logistics for the workshops and the unit’s education writers and clinician educators provided editorial support and content expertise to advance the development of the discipline RCEPAs, assessment strategies, competencies, training experiences, and standards of accreditation. The standardization of the series of activities and the Royal College support for the workshops and standards development was intended to address the stakeholder concerns related to workload, resources, and timelines.

## Learning Along the Journey

The design and delivery of the workshops were modified in response to feedback from specialty committee members, local and national program evaluation efforts, the observations of the Royal College team facilitating the workshops, and the realities of the COVID-19 pandemic.

The original design envisaged a series of two workshops, each three days long, focusing on curriculum design and learner assessment respectively. The first workshop began by guiding the specialty committee through a revision of the competencies of the discipline, including updates to the format, content, and language of CanMEDS 2015. The group started by rewriting the overall competencies of the discipline and moved on to write the developmental milestones for each stage of the Competence Continuum. Creating RCEPAs for the discipline was the focus of the third day. At the second workshop, the group refined the RCEPAs, wrote their assessment plans, and identified the training experiences a program was required to provide.

The original workshop plan was modified after experience with the first two disciplines, Medical Oncology and Otolaryngology — Head and Neck Surgery. Both the Royal College team and the specialty committee members reported that writing competencies and milestones before making other CBD decisions was difficult. Instead, clinicians found it easier to identify the EPAs of the discipline as this activity was more attuned to the way they thought about their work and postgraduate training. This sequence also facilitated the identification of the competencies required to perform those tasks. This feedback concurred with published reports [10,11,12] of the challenges clinicians face translating their work and their skills into the language of formal competency frameworks.

Early experience also guided the timing and sequencing of faculty development. The workshops included sessions aimed to help the specialty committee members prepare their programs for CBD implementation. Early feedback identified a need to appropriately sequence implementation activities to balance the volume and complexity of the change activities with front-line teachers’ capacity for change and the timing of their application of the new approaches to teaching and assessment.

These early experiences also identified that two workshops did not provide enough time to complete the work of SED.

The CBD workshop plans were revised to incorporate this feedback, becoming a series of 3 three-day workshops (see Table 2).

**Table 2:** The workshops to create the specialty educational design.


DURATION	WORKSHOP 1	WORKSHOP 2	WORKSHOP 3

	3 DAYS	3 DAYS	3 DAYS

Participant development	What is CBD? What are EPAs?	What is work based assessment? What are competence committees?	What is coaching? How are milestones used in CBD?

Standards development	Scope of practice Outcomes of training Progression through stages First draft of RCEPAs and training experiences	Refined RCEPAs Assessment requirements for RCEPAs Review Competencies document	Refined assessment plans for RCEPAs Milestones linked to RCEPAs Final revisions to Competencies, Training Experiences, and Standards of Accreditation

Preparation for implementation	Build awareness of the coming change Build local change management team	Establish a competence committee	Create curriculum map Create strategic plan for local implementation Orient front-line teachers and residents


Each workshop was a combination of activities including professional development regarding elements of Competence by Design (CBD), creation or revision of the educational standards for the discipline, and guidance about preparing for local implementation. *RCEPAs* – stage specific Royal College entrustable professional activities, *Competencies, Training Experiences, and Standards of Accreditation –* documents elaborating the discipline-specific Royal College educational standards. *Abbreviations:* CBD = Competence by Design; EPA= entrustable professional activity; RCEPA = Royal College approach to EPAs.

- The **first workshop** focused on delineating the scope of the discipline and the desired outcomes of training, establishing the sequential progression of trainee responsibility through the stages of the competence continuum, producing the first draft of specialty-specific RCEPAs, and establishing the required training experiences for each stage.
- The **second workshop** was used to refine the RCEPAs and develop assessment plans and the guidelines that competence committees would use when determining if the RCEPA had been achieved. The work of developing and refining the RCEPAs triggered rich discussions among the members of the specialty committee about the skills needed to perform the task. The Royal College team of a clinician educator and a writer used material from those discussions to draft milestones and competencies for the specialty committee to review at or before the third workshop.
- The purpose of the **third workshop** was to finalize all aspects of the SED, including the set of RCEPAs with assessment plans and milestones, and the discipline standards including the competencies, training experiences and standards of accreditation.

Between workshops, the Royal College writer and clinician educator supporting the group reviewed and revised the output of the workshops and engaged the workshop participants in clarification and advancement of their work.

Preparations for CBD implementation were sequenced: how to create awareness of the change was discussed at the first workshop, how to establish and run a competence committee at the second workshop, and how to create a curriculum map and strategic plan for local CBD implementation at the third. Additional webinars were timed to occur in the six months preceding CBD implementation and focused on how to orient front-line teachers and incoming trainees.

The onset of the COVID-19 pandemic required that the workshops be modified for delivery in a virtual setting; the sequence of activities was maintained. Over the two-year period in which the Royal College did not host in-person meetings, this shift to virtual workshops permitted 14 disciplines to complete their CBD education design and four others to begin the work; five disciplines held all their workshops in a virtual format. Specialty committee members were however eager to return to in-person meetings; a survey of workshop participants identified reasons for their preference, including the benefits of networking with each other, building a community of practice, and avoiding workplace distractions.

Finally, important modifications to elements of the CBD design were made based on feedback from the early cohorts of disciplines and local and national program evaluation efforts. Changes were made to the structure of the RCEPAs, to the format of the CanMEDS content, and to the selection of assessment strategies. Initial cohorts produced a large number of RCEPAs; upon implementation, this large number was a challenge for trainees, supervisors, and competence committees to manage and track. Subsequently, development of RCEPAs was focused on essential tasks of the discipline, and context menus were incorporated to allow for an RCEPA to be observed across a variety of its presentations (e.g., different settings such as clinic, ward, or emergency department, different ages such as child, adult, or elderly, or different conditions such as cardiac, respiratory, or renal); this reduced the typical number of RCEPAs to 30–50 per discipline. In addition, developmental milestones in the form of short CanMEDS competency statements had been linked to the RCEPAs as a prompt for supervisors to coach trainees on the skills related to the performance of that activity. Feedback identified that these milestones needed to be concise to work well on the digital platforms on which the assessments were completed; thus, the Royal College limited the number of milestones as well as their wordiness (see Supplementary file 2: Examples of RCEPA and milestone modifications). Furthermore, it was found that multisource feedback methods, patient inputs, and multi-part assessment tools were too complex to be executed given the current environment and digital platforms; in response, the RCEPA assessment strategies were adjusted. In this manner, the experience of the early-cohort disciplines informed important iterative changes.

## Identifying Benefits and Challenges of the Cohort Approach

The Royal College was both criticized and praised for its decision to spread and sequence the implementation of CBD over a decade through the cohort approach. Several key benefits of this approach have already been elucidated: modifying and adapting the workshops on the basis of early feedback; spreading the financial impact over time; and providing improved support to committees.

The cohort approach also provided time for the development of expertise within the Royal College team supporting the efforts of the specialty committees. While each committee only went through the SED process once, the Royal College team of clinician educators, writers, and administrators had repeated exposures to the process and experiences with a variety of medical, surgical, and diagnostic disciplines. This facilitated the Royal College’s ability to streamline the work for the committees by sharing relevant work from previous cohorts and standardizing some aspects of the CBD standards that were similar across disciplines (e.g., standard wording for an EPA about leading an inpatient service).

However, the lengthy approach to CBD transformation extended the period in which schools and programs had to manage the previous system of training simultaneously with the transformation to CBME. To some extent this was inevitable as the new training paradigm would apply only to incoming residents and not to those already training in multi-year programs; however, the decision to sequence CBD transformation across cohorts of disciplines prolonged the duration of the impact. This created challenges when trainees following one educational paradigm interacted with programs on the other educational paradigm. For example, when CBD trainees did “off-service” rotations in a discipline that had not yet started using CBD, those supervisors were not familiar with the use of RCEPAs and the assessment platform. Another example is resident transfer from one specialty to another and the challenges in applying credit for their training to date when the educational paradigms were using different measures of progress (i.e., successful completion of rotations versus documentation of RCEPA achievement and competence progression). The extended duration of implementation also meant schools had to manage two sets of standards during accreditation reviews with some programs following the “old” requirements and some following the new CBD requirements.

The decade-long process and normal turnover of specialty committee volunteers also meant that there were membership and leadership changes in the specialty committee which impacted “corporate memory” of decisions and plans before CBD was fully implemented in that discipline. Lastly, as more disciplines reached the stage of CBD implementation, the Royal College’s attention, resources, and capacity were divided between supporting the specialty committees that were in the process of implementation and supporting those that were still engaged in, or were waiting to engage in, their workshop series.

There were some unexpected delays in the timeline for CBD implementation. With the first three cohorts, the schools’ readiness limited the ability to move forward with implementation. As a result of this delay, some of the early-cohort disciplines disengaged from the change process, resulting in further challenges with program and faculty readiness. As the volume of disciplines engaged in the workshops increased, challenges in expanding the Royal College team supporting the initiative, in addition to turnover of clinician educators and education writers in the team, limited the Royal College’s capacity to continue with the planned rollout; the timelines for later cohorts were modified to allow for catch-up and to ensure ongoing sustainability of the initiative. The COVID-19 pandemic led to a six-month hiatus in the delivery of workshops as specialty committee members prioritized adapting their clinical practice to the realities of the changes in health care needs and delivery as well as the needs of their trainees; the Royal College shifted to a virtual working environment. The availability and/or willingness of disciplines heavily impacted by the pandemic (e.g., Public Health and Preventive Medicine) to engage in workshops and CBD implementation led to further delays in the cohort timeline.

## Achieving the Goal

The decisions taken by the Royal College in the road map for transformation enabled the volume of work required to develop training standards within the parameters CBD educational design. A small team of clinician educators, writers, and administrators was able to facilitate over 250 workshops with specialty committee volunteers. The CBD educational design was successfully adapted to meet the needs of training in a wide range of medical, surgical, and diagnostic specialties and subspecialties.

The first CBD workshop was held in September 2014 with Medical Oncology. The first disciplines to formally implement their CBD educational design into their training programs, in July 2017, were Anesthesiology and Otolaryngology — Head and Neck Surgery. As of July 2023, 65 (out of 67) specialties, subspecialties, and special programs have engaged in the transformation to CBD. Of those 65 disciplines, 61 have completed the creation of their specialty’s education design, 53 have implemented CBD in their training programs, and 24 have had trainees graduate after training in this paradigm.

## Conclusion

This paper describes the approach taken by the Royal College to launch a transformative change initiative that has reshaped the Canadian postgraduate specialty medical education system. We have characterized the considerations involved in planning this approach, delineated the principles that guided decisions about how to undertake this change, and described the impact of those decisions on the process and its outcomes to date. Other jurisdictions planning a change to CBME or any major initiative within the complex PGME environment may wish to consider:

- Incorporating principles and practices of change management at the onset and throughout the process, including engaging with partners early and often.
- Explicitly deciding whether to implement the initiative simultaneously across all parts of the PGME system, or sequentially. Each approach has its merits; we have delineated some of the benefits and challenges of the Royal College decision to sequence implementation.
- If a sequential approach is chosen, purposefully select the sequence of involvement (e.g., early adopters first, mix of disciplines).
- Systematically incorporating continuous quality improvement into the process; this includes establishing methods to collect feedback and identifying and interpreting intended and unintended consequences to enable adaptation.
- Involving content experts as well as those with expertise in the process. In our case, the specialty committee provided the content expertise for their discipline while the Royal College clinician educator and writer provided expertise in the CBD education design. The combination of expertise streamlined the work, ensured alignment with the CBD education design, and provided consistency and coherency across the 67 disciplines.
- Balancing strict adherence to the educational design with flexible adaptation for different disciplines or local contexts; shared common elements can facilitate sharing of resources, best practices, and lessons learned.

## Disclaimer

The views and opinions expressed in this article are those of the authors and do not necessarily reflect the official policy or position of the Royal College of Physicians and Surgeons of Canada (“Royal College”). Information in this article about Competence by Design (“CBD”), its implementation and related policies and procedures do not necessarily reflect the current standards, policies and practices of the Royal College. Please refer to the Royal College website for current information.

## Additional Files

The additional files for this article can be found as follows:

10.5334/pme.957.s1 Supplementary File 1: Appendix. Sequence and grouping of the cohorts.

10.5334/pme.957.s2 Supplementary File 2: Appendix. Examples of RCEPA and milestone modifications.
