# Supplementary material for: Competency-Based Medical Education at Scale: A Road Map for Transforming National Systems of Postgraduate Medical Education
Source: Perspect Med Educ. 2024 Feb 6;13(1):24–32. doi: 10.5334/pme.957 (PMC10870941; doi:10.5334/pme.957)
Supplement: Supplementary File 1: Appendix. — Sequence and grouping of the cohorts. [file pme-13-1-957-s1.pdf]

Supplementary file 1: Appendix. Sequence and grouping of the cohorts*

Cohort 1 (2014)	Cohort 2 (2015)	Cohort 3 (2016)	Cohort 4 (2017)	Cohort 5 (2018)	Cohort 6 (2019)	Cohort 7 (2020)
Medical Oncology	Anesthesiology	Anatomical Pathology	Cardiology	Adolescent Medicine	Dermatology	Colorectal Surgery
Otolaryngology — Head and Neck Surgery	Forensic Pathology	Cardiac Surgery	Clinical Immunology and Allergy	Child and Adolescent Psychiatry	Diagnostic Radiology	Developmental Pediatrics
	Gastroenterology	Critical Care Medicine	General Surgery	Clinical Pharmacology and Toxicology	Gynecologic Oncology	Endocrinology and Metabolism
	Internal Medicine	Emergency Medicine	Geriatric Medicine	Forensic Psychiatry	Gynecologic Reproductive Endocrinology and Infertility	General Surgical Oncology
	Surgical Foundations	General Internal Medicine	Neonatal-Perinatal Medicine	Geriatric Psychiatry	Infectious Diseases	Interventional Radiology
	Urology	General Pathology	Nuclear Medicine	Hematological Pathology	Maternal-Fetal Medicine	Neuroradiology
		Nephrology	Obstetrics and Gynecology	Hematology	Medical Biochemistry	Occupational Medicine
		Neurosurgery	Physical Medicine and Rehabilitation	Neurology	Medical Genetics and Genomics	Pain Medicine
		Pediatrics	Plastic Surgery	Neuropathology	Medical Microbiology	Palliative Medicine
		Radiation Oncology	Psychiatry	Orthopedic Surgery	Ophthalmology	Pediatric Radiology
			Respirology	Pediatric Hematology/ Oncology	Pediatric Emergency Medicine	Thoracic Surgery
			Rheumatology	Pediatric Surgery	Public Health and Preventive Medicine	
				Vascular Surgery		

The information above reflects when disciplines began the cohort transformation process.
*This initial rollout plan was modified over time due to the impact of resource limitations and the COVID -19 pandemic
