# Supplementary material for: Competency-Based Medical Education at Scale: A Road Map for Transforming National Systems of Postgraduate Medical Education
Source: Perspect Med Educ. 2024 Feb 6;13(1):24–32. doi: 10.5334/pme.957 (PMC10870941; doi:10.5334/pme.957)
Supplement: Supplementary File 2: Appendix. — Examples of RCEPA and milestone modifications. [file pme-13-1-957-s2.pdf]

Supplementary file 2: Appendix. Examples of RCEPA and milestone modifications

Comparison of a sample of RCEPAs from the Core stage of training in Otolaryngology-Head and Neck Surgery (Oto-HNS) with one from the same stage in Cardiac Surgery demonstrating adaptations in the approach to creating EPAs in response to feedback that having a large number of EPAs was problematic to implement. In both disciplines, this professional activity involves performing a clinical assessment and developing a management plan. Oto-HNS chose to write an EPA for each different presentation resulting in a large number of EPAs; Cardiac Surgery used context menus on the assessment form to collect information on different presentations and thus was able to combine a wide variety of presentations within just one EPA.

Sample RCEPAs from Oto-HNS Core stage	Cardiac Surgery Core EPA
1. Assessing and managing patients with non-neoplastic salivary disorders<br>2. Assessing patients with dysphagia or swallowing disorders<br>3. Assessing patients with facial paralysis, and providing recommendations for both surgical and non-surgical treatment options<br>4. Assessing and managing patients presenting with rhinosinusitis<br>5. Assessing and managing patients presenting with a sinonasal mass<br>6. Assessing and managing patients with nasal obstruction and/or septal deformities<br>7. Assessing patients with chronic airway obstruction<br>8. Assessing patients with dysphonia<br>9. Assessing and managing patients with mucosal squamous cell carcinoma of the head and neck<br>10. Assessing and managing patients with disorders of the thyroid glands<br>11. Assessing and managing patients with disorders of the parathyroid glands<br>12. Assessing and managing patients with neoplastic disorders of the salivary glands<br>13. Assessing and managing patients with head and neck surgical defects<br>14. Assessing and managing patients with benign or malignant skin lesions of the head and neck<br>15. Assessing and managing patients following facial trauma	Providing surgical consultation for patients with common presentations relevant to Cardiac Surgery<br><br>Form collects information on:<br>- Location: outpatient; inpatient<br>- Condition: coronary disease; aortic valve disease; mitral valve disease; tricuspid valve disease; aortic disease; pericardial disease; atrial myxoma; uncomplicated atrial septal defects/patent foramen ovale; arrhythmias requiring pacemaker insertion or surgical ablation; left ventricular outflow obstruction; acute pulmonary embolus<br><br>Collect at least 16 observations of achievement<br>- At least 4 outpatient<br>- At least 2 each of coronary disease; aortic valve disease; mitral valve disease; tricuspid valve disease; arrhythmias requiring pacemaker insertion or surgical ablation<br>- At least 1 each of aortic disease; pericardial disease; atrial myxoma; uncomplicated atrial septal defects/patent foramen ovale; left ventricular outflow obstruction; acute pulmonary embolus

Comparison of milestones associated with similar EPAs from the Core stage of training in Medical Oncology and Pediatric Hematology-Oncology (Peds Hem/Onc). Medical Oncology was one of the first disciplines to develop their RCEPAs and was coached to use milestones as they were written within the CanMEDs 2015 framework; Peds Hem-Onc adopted lessons learnt from the early cohorts to reduce the number of milestones and simplify and abbreviate milestones for better presentation on the electronic platform. The designation with the milestone denotes its CanMEDS role and location within the CanMEDS 2015 framework, i.e. ME is Medical Expert, COM is Communicator, COL is Collaborator, HA is Health Advocate, S is Scholar.

Medical Oncology: Core EPA #1	Pediatric Hematology-Oncology: Core EPA #1
Assessing new patients seen in consultation and planning management<br><br>1. ME 1.3 Apply knowledge of the clinical and biomedical sciences to the diagnosis and management of the clinical problem<br>2. ME 1.4 Perform comprehensive clinical assessments of patients presenting with the full range of presentations in Medical Oncology<br>3. ME 2.2 Synthesize and interpret investigations, and order additional investigations required to complete staging and provide additional information needed to make treatment decisions<br>4. ME 2.4 Develop and implement evidence-based and patient-centred management plans aligned with the goals of care<br>5. COM 3.1 Convey complex or sensitive information regarding diagnosis, prognosis, plan of care, (including risk, benefits, rationale, and alternatives to treatment) or uncertainty in a clear, compassionate, respectful, and accurate manner to the patient and family<br>6. HA 1.1 Advocate effectively for individual patients to help them overcome barriers to accessing the most effective evidence-based therapies, and receive the health services they need to effectively treat their cancer<br>7. HA 1.3 Identify opportunities for testing for familial or hereditary disposition to cancer<br>8. ME 2.4 Identify patients that may be eligible for clinical trials	Developing and implementing treatment plans for patients with pediatric hematology and oncology conditions<br><br>1. ME 2.2 Integrate the clinical data to establish the diagnosis and, if relevant, stage and/or severity of the condition<br>2. ME 2.3 Establish goals of care in collaboration with patients and their families<br>3. ME 2.4 Identify the role and availability of clinical trials participation as a therapeutic option<br>4. S 3.4 Apply an evidence-based approach to clinical management<br>5. ME 2.4 Develop a management plan<br>6. COL 1.2 Work effectively with members of the interprofessional team to implement a management plan

Copyright © 2018 The Royal College of Physicians and Surgeons of Canada. Referenced and produced with permission.
